# Genetic Diversity and Connectivity in the Threatened Staghorn Coral (*Acropora cervicornis*) in Florida

**DOI:** 10.1371/journal.pone.0008652

**Published:** 2010-01-11

**Authors:** Elizabeth M. Hemond, Steven V. Vollmer

**Affiliations:** Marine Science Center, Northeastern University, Nahant, Massachusetts, United States of America; American Museum of Natural History, United States of America

## Abstract

Over the past three decades, populations of the dominant shallow water Caribbean corals, *Acropora cervicornis* and *A. palmata*, have been devastated by white-band disease (WBD), resulting in the listing of both species as threatened under the U.S. Endangered Species Act. A key to conserving these threatened corals is understanding how their populations are genetically interconnected throughout the greater Caribbean. Genetic research has demonstrated that gene flow is regionally restricted across the Caribbean in both species. Yet, despite being an important site of coral reef research, little genetic data has been available for the Florida *Acropora*, especially for the staghorn coral, *A. cervicornis*. In this study, we present new mitochondrial DNA sequence data from 52 *A. cervicornis* individuals from 22 sites spread across the upper and lower Florida Keys, which suggest that Florida's *A. cervicornis* populations are highly genetically interconnected (F_ST_ = −0.081). Comparison between Florida and existing mtDNA data from six regional Caribbean populations indicates that Florida possesses high levels of standing genetic diversity (h = 0.824) relative to the rest of the greater Caribbean (h = 0.701±0.043). We find that the contemporary level of gene flow across the greater Caribbean, including Florida, is restricted (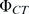 = 0.117), but evidence from shared haplotypes suggests the Western Caribbean has historically been a source of genetic variation for Florida. Despite the current patchiness of *A. cervicornis* in Florida, the relatively high genetic diversity and connectivity within Florida suggest that this population may have sufficient genetic variation to be viable and resilient to environmental perturbation and disease. Limited genetic exchange across regional populations of the greater Caribbean, including Florida, indicates that conservation efforts for *A. cervicornis* should focus on maintaining and managing populations locally rather than relying on larval inputs from elsewhere.

## Introduction

Coral reefs have declined rapidly over the past three decades, due in large part to the loss of dominant reef-building corals [1], [2]. A major factor contributing to the decline of coral reefs has been the rise in coral diseases, particularly in the Caribbean, which is now regarded as a “disease hot spot” [3], [4]. White band disease (WBD), in particular, has transformed Caribbean reefs by causing an unprecedented die-off of the two dominant shallow-water Caribbean corals, the staghorn coral (*Acropora cervicornis*) and the elkhorn coral (*A. palmata*). Since WBD was first observed in the late 1970s, record losses of up to 95% of live *Acropora* cover have been observed throughout the Caribbean [5], [6], and recovery has been slow to non-existent at most locations over the past two decades [7](but see [8], [9]). As a result, both species have been listed as threatened under the U.S. Endangered Species Act [10], [11] and as critically endangered under the International Union for the Conservation of Nature (IUCN) Red List criteria [12].

One key to designing appropriate management strategies and conserving the remaining Caribbean *Acropora* is knowledge about the extent to which populations of each species are interconnected via larval dispersal. Genetic exchange over large spatial scales (i.e. hundreds of kilometers) might allow distant healthy populations to rescue damaged reefs; whereas, restricted gene flow would indicate that populations rely on local recruitment and require local management. Information about the genetic make-up of Caribbean *Acropora* populations is also important since both species rely heavily on asexual fragmentation to propagate locally [13], [14] but must reproduce sexually during yearly mass spawning events to produce dispersing larvae [15], [16]. Because both species are largely self-incompatible ([17], Fogarty N, Vollmer SV, unpublished data), successful sexual reproduction requires that multiple genets are present and spawn on a reef. While genetic surveys indicate that multiple genets are often present in stands of both species [18], [19], it is unknown if small remnant *Acropora* populations have too few individuals to spawn consistently and successfully. The genetic make-up of *Acropora* populations may also affect their resiliency. For example, recent research indicates that 6% of *A. cervicornis* individuals are resistant to WBD [18], suggesting that populations with higher frequencies of resistant individuals may be more sustainable.

Recent genetic research on both Caribbean *Acropora* species indicates that gene flow is geographically restricted among populations separated by 500 km or more [20], [21]. Microsatellite data further indicate that *A. palmata* can be subdivided into distinct Western and Eastern Caribbean subpopulations [20], [22], and mitochondrial and nuclear sequence data from *A. cervicornis* detected fine-scale genetic differences among populations separated by as little as 2 km [21]. Regionally restricted gene flow in the Caribbean *Acropora* argues for regionally-based management [20], [21], but evidence for additional fine-scale differentiation in *A. cervicornis* suggests that the scale of dispersal and thus management at some locations may need to be much smaller (i.e. on the order of individual reefs) in this species [21]. Genetic studies of Indo-Pacific Acroporids have found evidence for population structure as well, but at a much larger geographic scale and generally of a smaller magnitude [23], [24] (but see [25], [26]).

One area where population genetic information from the Caribbean *Acropora* is lacking is the Florida Keys reef tract, which is the largest continuous barrier reef in the U.S. and a focal point for U.S. coral research in the Caribbean. The Florida Keys reef tract sits downstream of most Caribbean reefs, which makes it a possible sink for immigrant larvae from upstream source populations [27]. The predominant currents influencing larval transport into Florida's reefs are the Florida Current and the Loop Current, which is derived from the Caribbean Current after its passage between the Yucatan peninsula and Cuba. Oceanographic models indicate that northern Central American and Cuban reefs are the most likely sources for larval immigration into Florida, although larval exchange between the Bahamas and Florida is also possible [27], [28]. Microsatellite data from *A. palmata* support the genetic relationship between Florida and Western Caribbean, clustering Florida in the Western Caribbean subpopulation with Panama and Mexico, as well as with the Bahamas [20]. DNA sequence data for *A. cervicornis* also suggest a genetic connection between the Western Caribbean and Florida, but too few samples (n = 5) were available to estimate the extent of population genetic structure between Florida and the greater Caribbean.

To date, the five genets from Florida used for the study by Vollmer and Palumbi (2007) represented the entirety of our knowledge about the genetic state of this *A. cervicornis* population. Florida is at the northernmost limit of this species in the Caribbean [29], and Florida's *A. cervicornis* have a sparse and patchy distribution and have been heavily impacted by WBD [6], [30]. Due to Florida's location upstream of many other Caribbean reefs, genetic diversity of these corals might be influenced, and possibly increased, by receipt of immigrant larvae from upstream spawning populations [27]. However, within the Florida Keys, exposure to differing environmental factors may contribute to isolation of its populations. For example, the middle Keys reefs are exposed to higher inputs of water from Florida Bay, while the upper Keys, closer to the mainland, are subjected to more intensive terrestrial and anthropogenic influences [31]. If local recruitment is the primary source of *A. cervicornis* throughout the Keys, which is possible given the observation of genetic isolation over distances as short as 2 km in other *A. cervicornis* populations [21], we would expect to see genetic differentiation within the 200 km long Florida Keys reef tract.

Here we use mitochondrial DNA sequence data from 52 *Acropora cervicornis* individuals from 22 sites across the Florida Keys to evaluate the population genetic structure within the Florida Keys and compare these data to published sequence data from across the greater Caribbean. Genetic comparisons between Florida and the rest of the Caribbean allow us to estimate genetic connectivity within Florida and between Florida and the greater Caribbean and to evaluate the genetic diversity within Florida relative to other Caribbean populations. Based on oceanographic models and genetic data from *A. palmata*, we hypothesize that the Florida Keys reef tract is a sink for *A. cervicornis* larvae and genetic diversity from upstream sources, predominantly the Western Caribbean. We also evaluate the possibility of local recruitment and genetic structure within the Florida Keys reef tract, given previous observations of genetic structure in *A. cervicornis* over distances as small as 2 km [21].

## Results

**Figure 1:**
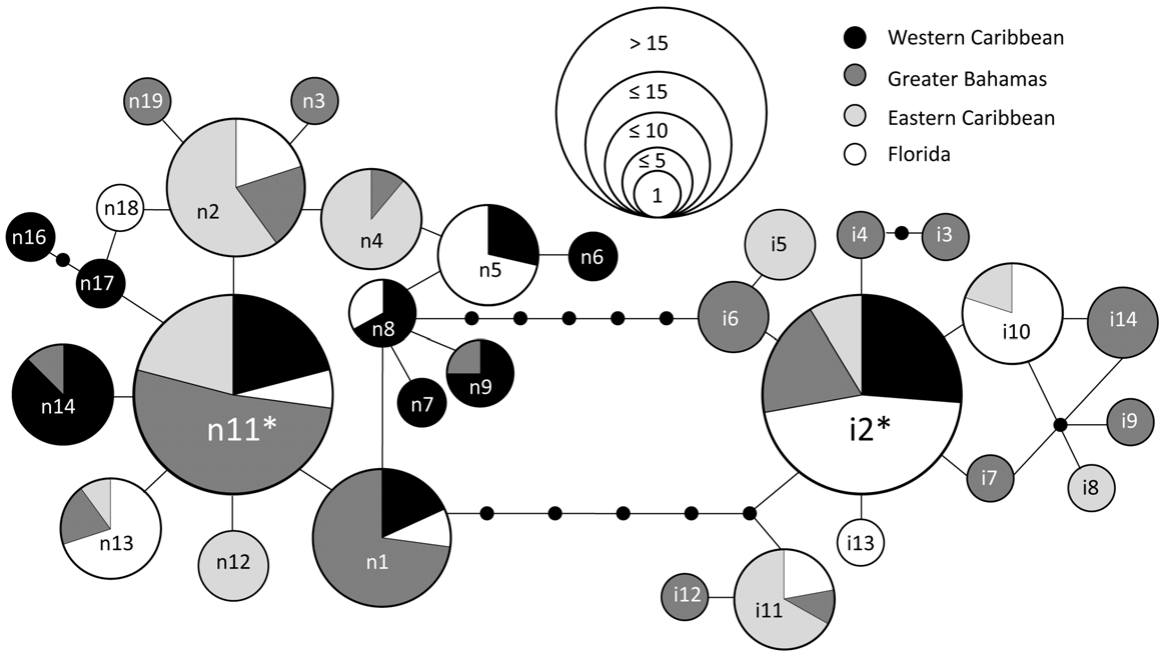
Haplotype network of native (n1 – n19) and introgressed (i1 – i14) mtDNA haplotypes found in Florida, Western Caribbean, Greater Bahamas, and Eastern Caribbean regions. The haplotype sequences have been submitted to GenBank under accession numbers GQ863966-GQ863998. Circle is drawn proportional to the number of times each haplotype was observed in the Caribbean. * Indicates haplotype designated as *ancestral* in TCS network.

Fifty-two control region sequences were produced for the Florida Keys, including 22 representing the upper Keys region, and 30 representing the lower Keys region. In addition, two sequences from Florida and 146 sequences from six regional populations throughout the Caribbean from Vollmer and Palumbi (2007) (Table 1) were used in the population genetic analyses. Thirty unique mtDNA control region haplotypes were observed in the Caribbean-wide sample (Figure 1, Table 2). Seventeen haplotypes are native to *A. cervicornis*, whereas 13 haplotypes represent introgressed haplotypes from *A. palmata* (resulting from interspecific hybridization [21], [32]). Seven native and four introgressed haplotypes were observed in the Florida Keys. The four native and two introgressed haplotypes found in the upper Keys were also found in the lower Keys, and an additional three native and two introgressed haplotypes were present in the lower Keys sample.

**Table 1:** Diversity values for native haplotypes of an 814 basepair fragment of *A. cervicornis* putative Control Region.

Population	N_t_	%I	N_n_	S	#hap	h	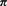	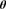 seq	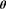 site	Tajima D	Fu & Li D	Fu & Li F	R_2_
Puerto Rico	26	27	19	3	4	0.678	0.00128	0.85834	0.00105	0.57845	1.01467	1.02929	0.1735
Curacao	19	42	11	3	3	0.618	0.00107	1.02425	0.00126	−0.50634	−0.87363	−0.88004	0.2096
Belize	20	0	20	10	8	0.847	0.00284	2.8187	0.00346	−0.63045	−0.96607	−1.00748	0.1099
Panama	25	60	10	4	4	0.800	0.00197	1.41394	0.00174	0.50521	1.23914	1.1866	0.2000
Bahamas	32	47	17	4	5	0.574	0.00094	1.18318	0.00145	−1.08236	−0.66882	−0.8952	0.1042*
Turks & Caicos	32	19	26	7	8	0.686	0.00135	1.83440	0.00225	−1.23319	−1.40617	−1.57613	0.0763*
Florida	54	61	21	5	7	0.824	0.00242	1.38976	0.00171	1.26153	0.37162	0.71852	0.1924
Lower Keys	30	56	12	5	7	0.879	0.00236	1.6557	0.00193	0.83404	0.56268	0.71557	0.1944
Upper Keys	22	68	7	4	4	0.810	0.00211	1.63265	0.00201	0.23902	−0.06863	0	0.2259

*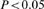.

Total number of sequences analyzed per population (N_t_), percent of total sequences found to be introgressed *A. palmata* haplotypes (%I), number of native haplotypes in the population used to calculate polymorphism statistics (N_n_), Length of sequences (L), number of polymorphic sites (S), Number of haplotypes, Haplotype diversity (h), Nucleotide diversity (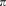), Theta per sequence (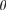
_seq_) and Theta per site (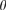
_site_) calculated from S, Tajima D, Fu & Li D, Fu & Li F. and R_2_.

**Table 2:** Native (n1-n19) and introgressed (i1-i14) haplotypes observed in each population in the Caribbean and in the upper (Up) and lower (Low) regions of the Florida Keys.

Haplotype	Panama	Belize	Bahamas	Turks & Caicos	Puerto Rico	Curacao	Florida	Total	Up	Low
n1	2		3	5			1	11		1
n2			1	2	3	6	3	15	2	1
n3			1					1		
n4				1	4	4		9		
n5	2						5	7	1	2
n6		1						1		
n7		1						1		
n8		2					1	3		1
n9		3		1				4		
n10								*		
n11	4	6	11	14	10		3	48	1	2
n12					2			2		
n13			1	1		1	7	10	3	4
n14	2	5		1				8		
n15								*		
n16		1						1		
n17		1						1		
n18							1	1		1
n19				1				1		
Total	10	20	17	26	19	11	21	124	7	12
i1								*		
i2	15		9	2	1	4	26	57	14	12
i3			1					1		
i4				1				1		
i5					2			2		
i6			2					2		
i7			1					1		
i8					1			1		
i9			1					1		
i10						1	4	5	1	3
i11			1		3	3	2	9		2
i12				1				1		
i13							1	1		1
i14				2				2		
Total	15	0	15	6	7	8	33	84	15	18

*Indicates a haplotype observed in Vollmer and Palumbi (2007) [21], but absent in this study.

Table 1 presents genetic diversity statistics for the native mtDNA haplotypes and includes the percentage of introgressed mtDNA haplotypes in each population sample. Florida had the highest proportion of introgressed haplotypes (61%) and no significant difference in introgression frequencies was detected between the lower and upper Keys (G = 0.26, df = 1, *P*>0.5). The high frequency of introgressed haplotypes in Florida was matched only by Panama, with an introgression frequency of 60%. Other Caribbean populations have introgressed haplotype frequencies between 19%–47%. Belize was an exception with no introgressed haplotypes (n = 20). Introgression frequencies of haplotypes varied significantly across the seven Caribbean samplings (G = 14.02, df = 6, *P*<0.05).

Nucleotide and haplotype diversity values for the native mtDNA haplotypes were similar among populations across the greater Caribbean (Table 1). Bahamas had the lowest haplotype and nucleotide diversity (h = 0.574 and 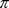 = 0.00094), whereas Florida (h = 0.824 and 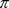 = 0.00242) and the two Central American populations (Panama, h = 0.800 and 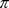 = 0.00197; Belize, h = 0.847 and 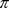 = 0.00284) had the highest values. Within Florida, although the upper Keys had fewer haplotypes (n = 4) than the lower Keys (n = 7), when adjusted for sample size, both had similar levels of haplotype and nucleotide diversity (upper Keys h = 0.810, 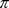 = 0.00211; lower Keys h = 0.879, 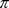 = 0.00236). Theta values were comparable across Caribbean populations, and neutrality tests (Tajima's D, Fu and Li's F and D, mismatch analysis and R_2_) do not suggest significant deviations from neutral expectations including population bottlenecks or expansions, except in the Bahamas and Turks and Caicos Islands where R_2_ was significant.

### Phylogeographic Distribution of mtDNA Haplotypes

The haplotype network shows the relatively high levels of mtDNA diversity of native (n) and introgressed (i) haplotypes and their distributions across the four Caribbean regions (Figure 1). For the native mtDNA haplotypes, a single haplotype (n11) was distributed across all four regions; this is the most likely ancestral native haplotype. Three native haplotypes were distributed across three regions; n1 was present in the Western Caribbean, Florida and Greater Bahamas, whereas n2 and n13 were present in Florida, the Greater Bahamas and Eastern Caribbean. The remaining 13 native haplotypes had geographically restricted distributions and were observed either in two geographic regions (five haplotypes: n4, n5, n8, n9, n14) or in a single region or population (eight haplotypes). Geographically restricted haplotypes tended to be more derived and should reflect contemporary genetic connections among regions. Of the five geographically restricted haplotypes shared across two of the four regions, one haplotype was shared between the Greater Bahamas and Eastern Caribbean (n4), two haplotypes were shared between the Western Caribbean and Greater Bahamas (n9 and n14), and two haplotypes were shared between the Western Caribbean and Florida (n5 and n8); the Western Caribbean and Eastern Caribbean lacked shared haplotypes. The remaining eight native haplotypes were observed only in a single region or population (i.e. private haplotypes). Four of these haplotypes were observed in Belize (n6, n7, n16 and n17), one in the Bahamas (n3), one in the Turks and Caicos (n19), and one in Florida (n18).

With respect to the phylogeographic distribution of the seven native haplotypes observed in Florida, four haplotypes had broad geographic distributions (n1, n2, n11, n13), whereas the remaining three haplotypes had geographically restricted distributions. Of the geographically restricted haplotypes found in Florida, which are likely to reflect recent gene flow, two were shared between Florida and the Western Caribbean (n5 and n8), providing evidence of a link between these two regions. The other haplotype was exclusive to Florida (n18).

Thirteen introgressed mtDNA haplotypes were detected in *A. cervicornis* across the Caribbean. Haplotype i2 was the most common introgressed haplotype and was found in all four regions; it is also the most common and ancestral haplotype in *A. palmata* [32]. Haplotype i2 was the only introgressed haplotype observed in the Western Caribbean. Two introgressed haplotypes were found in two (i10) or three (i11) regions, whereas the remaining 10 introgressed haplotypes were found in only one region and often only in one population, including one introgressed haplotype found only in Florida (i13). The relatively high proportion of private introgressed haplotypes most likely reflects both the rarity of local introgression events [32] and possibly the restricted geographic distribution of these haplotypes in *A. palmata*. Regarding the four introgressed haplotypes found in the Florida sample, two haplotypes had broad geographic distributions (i2 and i11), and two were restricted to one (i13) or two (i10) regions. Haplotype i10 is a restricted haplotype shared between the Eastern Caribbean and Florida; however, this could reflect gene flow in either *A. cervicornis* or *A. palmata*. Because of the high variance in introgression frequency across populations, the distribution of introgressed haplotypes may not accurately reflect gene flow among populations of *A. cervicornis*; rather, it may represent differential gene flow between *A. palmata* and *A. cervicornis* occurring in different regions.

### AMOVA and Population Genetic Structure

Within Florida, due to the small sample sizes within sites, AMOVA and analysis of population structure at the level of reefs were not possible. To evaluate gene flow among the Florida Keys, collection sites were classified as upper Keys or lower Keys according to geography (Figure 1). No evidence for significant population structure between the upper Keys and lower Keys in either the complete dataset (F_ST_ = −0.024, *P* = 0.66) or the native only dataset (F_ST_ = −0.081, *P* = 0.79) was observed, and both upper and lower Keys had similar levels of introgressed haplotypes. As a result, the upper Keys and lower Keys samples were combined into a single Florida population for the Caribbean-wide population genetic analyses.

Hierarchical AMOVA was used to compare the levels of population genetic differentiation among regions (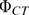), among populations (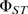), and among populations within regions (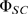). Both the complete dataset and native only dataset reveal strong and significant levels of genetic differences among populations (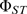, Table 3). For the native haplotypes, the AMOVA indicates that strong population genetic differences exist among the four regions, Western Caribbean, Eastern Caribbean, Greater Bahamas and Florida (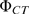 = 0.117, *P* = 0.02), but not among populations within regions (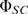 = 0.030, *P* = 0.11). In contrast, AMOVA for the complete dataset indicates structure among populations within regions (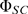 = 0.176, *P*<0.001) but not among regions (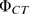 = −0.048, *P* = 0.66). This demonstrates that the inclusion of introgressed haplotypes in the analysis increases the variation among populations, but obscures the regional structure revealed in the native haplotypes.

**Table 3:** AMOVA results showing levels of genetic structure among regions (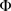
_CT_), among populations within regions (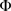
_SC_), and among populations (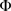
_ST_).

	Source of variation	df	Variance components	Variation (%)	Fixation Index	Significance (P)
All haplotypes	Among Regions	3	−0.11	−4.85	_ΦCT_ = −0.048	0.638
	Among Populations within Regions	3	0.42	18.43	_ΦSC_ = 0.176	0.000
	Within Populations	201	1.98	86.42	_ΦST_ = 0.136	0.000
	Total	207	2.29			
Native haplotypes	Among Regions	3	0.10	11.65	_ΦCT_ = 0.117	0.020
	Among Populations within Regions	3	0.02	2.62	_ΦSC_ = 0.030	0.113
	Within Populations	117	0.70	85.73	_ΦST_ = 0.143	0.000
	Total	123	0.82			

Data were analyzed separately for combined native and introgressed haplotypes and for native haplotypes only. Significance tests are based on 10,100 permutations.

Pairwise F_ST_ values show varying degrees of population genetic structure across the greater Caribbean in both the native and complete datasets. For native haplotypes, pairwise F_ST_ across all comparisons ranged from 0 to 0.477 (mean ± SE = 0.141±0.028). Curacao was the most distinct population with all pairwise comparisons being significant and four of six comparisons remaining significant after sequential Bonferroni correction. For the native dataset, significant population structure was observed between Florida and all other Caribbean populations (F_ST_ = 0.117±0.011), except Panama (F_ST_ = 0.042); however, after sequential Bonferroni correction, none of the comparisons remained significant (Table 4). For the complete dataset, pairwise F_ST_ across all comparisons ranged from 0 to 0.424 (mean ± SE = 0.133±0.027). For this dataset, Belize was the most distinct population with four of six pairwise comparisons being significant after Bonferroni correction, largely due to the absence of introgressed haplotypes in the Belize sample. In the complete dataset, significant population structure was observed between Florida and three of the four Caribbean populations, but after sequential Bonferroni correction only two comparisons are significant (Belize and Turks and Caicos).

**Table 4:** Pairwise F_ST_ between populations.

	Puerto Rico	Curacao	Panama	Belize	Bahamas	Turks & Caicos	Florida
Puerto Rico		0.007	0.186**	0.114**	0.060	−0.003	0.177**
Curacao	0.213*		0.094*	**0.246** **	0.020	0.078	0.079*
Panama	0.106	**0.338** **		**0.424** **	0.008	**0.233** **	−0.020
Belize	0.139**	**0.318** **	−0.032		**0.275** **	0.078*	**0.390** **
Bahamas	0.104*	**0.477** **	0.053	0.102*		0.091*	0.018
Turks & Caicos	0.074*	**0.385** **	−0.007	0.063*	−0.030		**0.223** **
Florida	0.099*	0.157*	0.042	0.106*	0.126*	0.097*	

*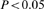,

**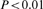,

bold = significant after sequential Bonferroni adjustment.

Upper right calculated for the combined native and introgressed haplotype dataset, and lower left calculated with native *A. cervicornis* haplotypes. Significance tests are based on 10,100 permutations.

## Discussion

Our results demonstrate that significant genetic structure exists among *Acropora cervicornis* populations across the greater Caribbean (native 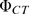), indicating that gene flow is restricted over regional scales (500 km or more). High levels of genetic differentiation detected in the native mtDNA haplotypes among the four Caribbean regions (native 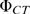 = 0.117) corresponds to a rate of inter-regional gene flow on the order of 3.8 migrants per generation across the greater Caribbean (after [33]). The relatively large proportion of native mtDNA haplotypes with restricted geographic distributions [i.e. in only one or two regions (47% and 29%, respectively)] provides additional support for the regional genetic structure detected with F-statistics and AMOVA and reflects the limits on gene flow over large spatial scales.

Our data indicate that Florida's *A. cervicornis* population is not genetically depauperate and has similar levels of genetic diversity to regions elsewhere in the Caribbean. We detected no evidence for population structure within Florida (between the upper and lower Keys) in our dataset. However, we detected relatively high levels of genetic structure between Florida and other populations in the Caribbean (average native pairwise F_ST_ = 0.105
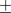
0.016). Panama was genetically most similar to Florida (native F_ST_ = 0.042), whereas Curacao was most different (native F_ST_ = 0.157). Native F_ST_ values for all other populations with Florida were between 0.097–0.126. Florida and the Western Caribbean share geographically-restricted haplotypes, suggesting that the Western Caribbean has historically been an upstream source of genetic diversity for Florida.

### Genetic Connections between Florida and the Rest of the Caribbean

Evidence presented here for restricted gene flow in *Acropora cervicornis* across large swaths of the Caribbean support the previous findings of Vollmer and Palumbi's (2007) multi-locus Caribbean-wide survey. In addition to the genetic structure among Caribbean populations observed in the previous study (Φ_CT_ = 0.249) and supported here (Φ_ST_ = 0.143), the data demonstrate significant structure among regional groupings (Western Caribbean, Eastern Caribbean, Greater Bahamas and Florida, Φ_CT_ = 0.117). Despite this population structure, analysis of shared haplotypes indicates a historical and possibly on-going connection between *A. cervicornis* populations in the Western Caribbean and Florida, which Vollmer and Palumbi (2007) tentatively suggested with a small sample of Florida *A. cervicornis* (a total of five genets). While Florida, the Greater Bahamas and the Eastern Caribbean also share some haplotypes, strong genetic connections are not evident between these regions. Although the Bahamas is much closer to the Florida Keys (200 km) than the other populations, the strong Gulf Stream current appears to act as a barrier to gene flow. Shared haplotypes between Florida and the greater Caribbean are likely to be the result of gene flow to Florida, rather than from Florida into the other Caribbean regions due to prevailing currents [34]. Yet, a protracted pelagic larval stage and favorable currents may allow for gene flow from the Florida Keys to the Bahamas [28].

Ocean currents support a link between the Western Caribbean and Florida [28], but coral reefs in Panama should be less interconnected with Florida than those of Belize based on dispersal routes and distances and the retaining influence of the Colombia-Panama Gyre (contrary to our data). In addition, the transfer of genetic variation between Central America and Florida (1000 km from Belize and 1900 km from Panama) would likely require multiple generations of dispersal in a stepping stone fashion via intermediate populations. With a relatively short larval stage (∼4 days, Vollmer SV, Fogarty N, unpublished data), the dispersal potential of *A. cervicornis* larvae should be on the order of tens of kilometers at most. Sharing of haplotypes between Western Caribbean and Bahamas (1200 km apart) may also be accomplished via a similar route of connectivity along the reefs of Cuba [28]. Larvae with such a short competency period still have a very low probability of surviving each leg of this journey. Thus, the phylogeographic connections in our data may reflect historical patterns of gene flow that occurred decades, centuries or longer ago in this species, which is both long-lived and able to propagate indefinitely through asexual reproduction [35].

Significant differences in the frequencies of introgressed haplotypes among populations provides another strong indication that gene flow among these populations is geographically restricted. With free exchange of larvae among populations, introgression frequencies would homogenize, but the data demonstrate that this is not the case. The extremely high introgression frequencies in Florida and Panama distinguish these populations from elsewhere in the Caribbean, including the Greater Bahamas. While the similarity in introgression frequencies between Florida and Panama may be due to ongoing gene flow, we consider this unlikely given the distance and genetic structure of both populations with Belize, an intermediate population. It is possible that *A. cervicornis* in Panama and Florida share similar characteristics that favor introgressive hybridization, although it is not clear what those characteristics might be. The rarity of *A. cervicornis* in Florida could increase the likelihood of inter-specific fertilization, but this cannot explain the high introgression frequency in Panama, which has dense thickets of *A. cervicornis*.

Given the relatively short dispersal potentials of the Caribbean *Acropora* coral (3–5 days) and reef corals in general [22], [36], [37], it is perhaps not a surprise that population genetic studies of a diversity of Indo-Pacific and Caribbean reef coral species indicate that gene flow tends to be restricted over hundreds of kilometers (summarized in [21]). Regionally restricted gene flow has been detected in both of the major reef-building Caribbean coral groups, *Acropora* [20], [21] and *Montastrea* corals [38]. Caribbean-wide population genetic analyses for *A. palmata* using microsatellites have shown strong genetic structure across the greater Caribbean with a genetic break between the Western Caribbean and the Eastern Caribbean occurring at the Mona Passage between Puerto Rico and the Dominican Republic [20], [22]. Genetic data from *A. palmata* support a connection between Florida and Western Caribbean reefs [20], [22] similar to *A. cervicornis* [21]. But unlike for *A. cervicornis*, in *A. palmata* there appears to be a strong genetic connection between Florida and the Bahamas [20], [22]. Microsatellites and RFLP analysis of two *Montastrea* species within the same genus revealed that one species, *M. annularis*, exhibits high population differentiation while the other, *M. faveolata*, appears panmictic between the Western Caribbean (Yucatan), Eastern Caribbean (Puerto Rico) and Florida [38]. Thus, in both the Caribbean *Acropora* and *Montastrea*, genetic data suggest that related coral species with similar life-histories and dispersal potentials can have contrasting population structures. Future research is needed to explain these differences.

Differing degrees of population genetic structure have also been detected in a variety of other Caribbean reef taxa and ascribed to a variety of causes. Within the Florida Keys, structure was observed in damselfish over a few meters and attributed to either a local genetic bottleneck [39] or recruitment from genetically divergent source populations, such as the Bahamas and Western Caribbean [27]. Across the Caribbean, the significant population genetic structure detected in fishes including gobies (*Elacatinus* spp) [40], damselfish (*Stegastes partitus*) [41] and wrasses [42] has been explained by isolation by distance [41] as well as differences in environmental factors [42]. Invertebrates have shown varying levels of genetic connectivity, from high genetic structure in an octocoral (*Pseudopterogorgia elisabethae*) [43], [44] to almost no genetic structure in the economically important species queen conch (*Strombus gigas*) [45] and spiny lobster (*Panulirus argus*) [46].

### Genetic Diversity and Connectivity within Florida's Staghorn Corals

Our data revealed no significant population structure among *A. cervicornis* within the Florida Keys. This is surprising, given that our samples were collected over a distance of 200 kilometers and a range of environmental conditions. Previous research on *A. cervicornis* by Vollmer and Palumbi (2007) detected multiple instances in which *A. cervicornis* populations separated by 2–15 kilometers were genetically distinct. In Florida, however, no population genetic differences were detected between the upper Keys and lower Keys in either native or introgressed haplotypes. The absence of population genetic structure within Florida may indicate that gene flow is high across the Florida Keys reef tract, but alternate explanations are possible. In particular, barriers to gene flow between reefs may exist at a small geographic scale, but due to the limited sampling available from this diminished *A. cervicornis* population such fine-scale analyses could not be applied. Similar haplotype frequencies between upper and lower Keys may also result from recent mortality due to WBD that may be exhibiting positive selective pressure on resistant genotypes [18] and reducing local diversity; however, our analyses indicate relatively high diversity along the Florida Keys as a whole. While it is possible that highly polymorphic genetic markers, such as microsatellites, might reveal additional population genetic structure within the Florida Keys, preliminary microsatellite data for *A. cervicornis* indicates that no such structure exists (I. Baums, personal communication). Thus, it may well be the case that Florida is characterized by having high gene flow within the region.

It is not entirely clear why Vollmer and Palumbi (2007) detected such fine-scale differences among reefs elsewhere. Much of the fine-scale genetic differences in their dataset were driven by highly localized introgression signatures at one or more of the mtDNA or nuclear intron loci surveyed, including the putative mtDNA control surveyed here. Florida is distinctive for having the highest frequencies of introgressed mtDNA haplotypes detected to date across the greater Caribbean, but interestingly these high frequencies do not differ between the upper and lower Keys (G = 0.26, df = 1, *P*>0.5). The absence of localized differences in introgression frequencies provides additional support for high gene flow across Florida Keys reef tract; however, the low abundance of *A. cervicornis* may have resulted in a higher proportion of hybrid recruits. Additional investigation of the geographic patterns of hybridization and introgression may shed light on this matter.

High gene flow across the Florida Keys is a possible indication that the genetic diversity present in this population is sufficient to allow sexual reproduction via outcrossing. Sufficient genetic diversity and larval recruitment are essential for recovery of at risk populations of corals, and our results indicate that Florida's genetic diversity of native haplotypes (h = 0.824) is comparable to, and even higher than, the rest of Caribbean (h = 0.701
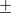
0.043). In addition, while a number of haplotypes are regionally restricted, Florida's *A. cervicornis* population contains haplotypes found in all other regions. Historical recruitment from the Western Caribbean and other regions is one possible explanation for the relatively high diversity in Florida.

The relatively high genetic diversity and the results of tests for population size fluctuations (Tajima's D, Fu & Li's D, Fu & Li's F, and R_2_) do not indicate that there has been a significant loss of gene diversity (i.e. a genetic bottleneck) associated with the recent declines of *A. cervicornis* in Florida due to WBD. This may not be a surprise given that it should take multiple generations of random genetic drift for population size reductions to be reflected in genetic diversity estimates [47], especially in large populations. This could take many years in a clonal species with an indefinite life-span. Even in species with short life spans, genetic diversity may not immediately reflect dramatic population size reductions. For example, Caribbean populations of the long-spined black sea urchin (*Diadema antillarum*), which suffered an analogous decline of up to 97% throughout the Caribbean as a result of disease in the early 1980s, also retained high genetic diversity in an mtDNA marker surveyed for individuals collected between 1987 and 1999 [48]. In both cases, it may take time before a genetic bottleneck is evident in genetic diversity and effective population size estimates.

The effective population size of *A. cervicornis* in Florida can be estimated using the estimated theta value from the native mtDNA diversity and the equation 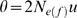 for mitochondrial DNA [where N*_e(f)_* is the effective number of females, which is equivalent to N*_e_* (the effective population size) because *A. cervicornis* is hermaphroditic, and *u* is the mutation rate per generation]. To determine the neutral mutation rate of the putative mitochondrial control region (*u*), we used the current estimated divergence time between *A. palmata* and *A. cervicornis* of 3.6–2.6 mya, which corresponds to 350,000 to one million generations ago using a generation time of 3–8 years [49]. Based upon the presence of six diagnostic mutations between *A. palmata* and *A. cervicornis* sequences of 814 bp, we estimated a neutral mutation rate between 2.106×10^−8^ and 7.371×10^−9^ mutations per basepair per generation. Given the estimated theta (per site) for Florida (0.00171), this corresponds to an estimated range of effective population size of 40,600–116,000 individuals within the Florida Keys.

This estimate is far less than the 2007 abundance estimates of Miller *et al.* (2008) [50], which indicate 13.8±12.0 million colonies of *A. cervicornis* in the Florida Keys. Clearly, more precise estimates of current census population sizes are needed. One reason for the discrepancy between census and effective population size estimates is that the effective population size reflects effective number of genets rather than ramets, and there are likely to be many ramets per genet in this asexually reproducing species. In addition, effective population size is often much smaller than the census size due to gender imbalance (not a factor in hermaphroditic species), variance in reproductive success [51], fluctuating population size, population subdivision with frequent extinction and recolonization [52] or a combination of these factors [53]. However, due to the widespread evidence of recent population decline of *A. cervicornis* and the tendency of effective population size estimates to reflect the long-term average (harmonic mean) population size [54], our effective population size estimate may not significantly underestimate the true census size of genetically distinct colonies of this species in the Florida Keys.

The high standing genetic diversity in the Florida Keys is a hopeful sign for future resilience of *A. cervicornis* along these reefs, but over time the effect of genetic drift in a small population may result in a future genetic bottleneck. Even with relatively high genetic diversity, successful reproduction will occur at the level of individual reefs and requires that multiple genotypes are present. Future research should address the extent of localized genotypic diversity on Florida Keys reefs with additional sampling and long-term monitoring.

### Conclusions

The significant levels of population structure detected between Florida and other regions in the Caribbean reveal that ongoing rates of recruitment to the Keys from reefs elsewhere are low. Restricted gene flow between Florida and other Caribbean populations indicates high dependence on local larval retention within the Florida Keys on the whole. Overall, our data suggest that the *A. cervicornis* in the Florida Keys comprise a unique population within the Caribbean and should be treated as a distinct management unit for conservation. Monitoring of genetic diversity should continue for the Florida Keys region as the effect of a genetic bottleneck may be lagging behind the observed decrease in population size resulting from WBD. Furthermore, the genetic make-up may shift if WBD takes a greater toll on disease susceptible genotypes [18] or if high recruitment of larvae occurs from elsewhere. Continued genetic analysis of additional samples, as they become available, will help to reveal the extent of local genotypic diversity and clarify whether barriers to gene flow exist between individual reefs in the Florida Keys. Current data showing limited genetic inputs from the greater Caribbean and gene flow within the Florida Keys suggest that the persistence of populations of this important reef-building species in Florida in the immediate future will depend on self-recruitment, and thus must be managed as a local resource.

## Materials and Methods

Collection of coral samples for this project was approved by the National Oceanic and Atmospheric Administration and was conducted under permit numbers FKNMS-2008-006 and FKNMS-2007-061.

### Sampling and Data Collection

**Figure 2:**
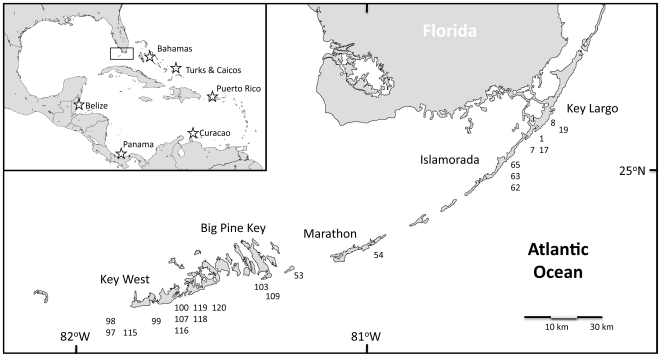
Sampling locations of *A. cervicornis*. Sampling sites in the Florida Keys and inset map of the greater Caribbean with sampling locations from Vollmer and Palumbi (2007). Numbers correspond to site names in Table 5. Rectangle in Caribbean map indicates the location of the Florida Keys.

For this study, 52 mtDNA sequences were produced from staghorn corals sampled from 22 populations spread across the Florida Keys. *A. cervicornis* specimens were collected July-September 2008 from 22 sites spanning the Florida Keys from southwest of Key West to Key Largo (Table 5, Figure 2), a distance approximately 200 km long. Each site represents a patch of *A. cervicornis* individuals. One to six corals were sampled per site (i.e. staghorn coral patch), which reflects the biological reality of the typically small patch sizes within Florida. Tissue samples were collected by sampling a small (1 cm) branch tip from staghorn coral colonies spaced at least 10 meters apart to minimize collection of clones produced by asexual fragmentation within each patch (Vollmer, in prep). Tissue samples were preserved in Chaos DNA extraction buffer and stored at room temperature. Extraction of DNA from the samples was conducted using a modified phenol-chloroform procedure [55]. The dataset used here also includes published data from Vollmer and Palumbi's (2007) Caribbean-wide population genetic survey of *A. cervicornis*, consisting of mtDNA sequence data from 148 individuals from six geographic regions plus Florida (Figure 2): the Bahamas (n = 32 individuals), Turks and Caicos (n = 32), Puerto Rico (n = 26), Curacao (n = 19), Belize (n = 12), Panama (n = 25), and Florida (n = 2). Eight additional sequences from Belize were also added to improve the previous sample size (from 12 to 20).

**Table 5:** Florida sampling locations listed roughly southwest to northeast.

Region	Site #	Site location	Lat. (N)	Long. (W)	*Acropora cervicornis* samples
Lower Keys	97	Middle Ground	24° 28.427′	81° 52.897′	3
	98	Middle Ground	24° 28.801′	81° 52.949′	2
	115	East of Eastern Dry Rocks SPA	24° 27.879′	81° 50.217′	1
	99	West of Western Sambo ER	24° 29.509′	81° 43.729′	2
	116	No Name Reef	24° 29.730′	81° 38.910′	1
	107	North of Pelican Shoal	24° 30.520′	81° 37.787′	4
	100	North of Eastern Sambo RO	24° 31.382′	81° 38.940′	4
	118	Pelican Shoal	24° 30.036′	81° 37.716′	3
	119	Maryland Shoal	24° 31.327′	81° 34.649′	1
	120	American Shoal	24° 31.383′	81° 31.190′	1
	103	North of Looe Key RO	24° 35.590′	81° 23.904′	2
	109	East of Looe Key RO	24° 34.367′	81° 22.922′	4
	53	South of Ohio Key	24° 37.637′	81° 13.872′	1
	54	South of Duck Key	24° 42.993	80° 56.224′	1
Upper Keys	62	North of Davis Reef	24° 56.895′	80° 29.843′	3
	63	North of Davis Reef	24° 57.241′	80° 29.775′	3
	65	North of Davis Reef	24° 57.410′	80° 29.603′	6
	7	Inshore of Pickles Reef	24° 59.549′	80° 25.860′	2
	17	Pickles Reef	24° 59.329′	80° 24.825′	1
	1	Inshore of Molasses Reef	25° 02.359′	80° 23.605′	1
	8	Inshore of French Reef	25° 03.169′	80° 21.766′	4
	19	North of French Reef SPA	25° 02.400′	80° 20.727′	2
Total					52

Sites 53–65 are technically middle Keys, but have been grouped by proximity into either upper Keys or lower Keys.

Population genetic analyses were conducted using the putative mitochondrial control region [56], a 941-bp fragment that has been shown to have high haplotype diversity and the ability to resolve population genetic structure in *A. cervicornis* across the Caribbean [21]. Three nuclear genes previously evaluated for *A. cervicornis* have few native alleles (2 in MiniCollagen and 1 in both Calmodulin and PaxC), and thus signatures of population structure are dominated by introgression [21]. Multiple microsatellite loci designed from *A. palmata* [17] are also available for *A. cervicornis*; however, preliminary analyses of these loci indicate that they are confounded by introgression and homoplasy (Vollmer, pers. obs.). In order to avoid the confounding effects of introgressed alleles, we used mtDNA sequence data, which allowed for identification of introgressed and native haplotypes. Polymerase chain reaction (PCR) amplifications and DNA sequencing of the putative mtDNA control region were carried out according to [32], and sequencing was performed on ABI sequencers (Applied Biosystems, Foster City, CA). Sequences were edited and aligned manually using Sequencher 4.8 (Gene Codes Corp., Ann Arbor, MI). Ends of the sequences were trimmed to a total sequence length of 814 base pairs. The region contained two informative insertion/deletion (indel) regions, which were coded as single base changes for population genetic analyses.

Due to the small sample sizes within Florida (n = 1–6 samples per site), it was not possible to examine population structure between each site, rather the sites were more broadly classified into two regions, upper Keys (n = 22) and lower Keys (n = 30), for analyses of structure within Florida (Table 5). Samples were assigned to these regions based on the natural break in the dataset due to a distance of 80 km between the most southwestern upper Keys site (62) and most northeastern of the clustered lower Keys sites (53) (Figure 2); the one sample from site 54 was grouped with the lower Keys due to proximity. Upper and lower regions of the Keys are subjected to different environmental conditions, including currents and proximity to terrestrial and anthropogenic influences. While lower Keys reefs run west to east and may be influenced by the Pourtales Gyre [57], the upper Keys have a more north-south orientation and primarily experience the northeastward flow of the Florida Current. In addition, Florida Bay water delivered to the reef tract through channels in the middle Keys may influence coral and larval survival and reef connectivity between upper and lower Keys as well. However, the population structure analysis detected no population structure within the Florida Keys reefs (see results), and hence all Florida samples were treated as a single population for the Caribbean-wide population genetic comparisons.

It has been shown that *Acropora cervicornis* hybridizes and exchanges genes with its congener *A. palmata* [32], [49], and that the pattern of this introgressive gene flow is one-way from *A. palmata* into *A. cervicornis* [21], [32]. This one-way gene flow allows for the identification of mtDNA haplotypes that are either introgressed (i.e. from *A. palmata*) or native to *A. cervicornis* [21], [32]. Vollmer and Palumbi (2007) have shown that including introgressed genes in population genetic analyses of *A. cervicornis* obscures native population structure across the Caribbean, but adds to the genetic structure between local populations (i.e. reefs) due to strong differences in introgression frequencies among local staghorn coral populations. To account for the differences between native mtDNA haplotype variation (i.e. reflecting intra-species gene flow only) and variation in the complete dataset including introgressed genes (i.e. reflecting intra- and inter-specific gene flow), we split the mtDNA data into two datasets for analyses: one complete dataset including all haplotypes (i.e. native and introgressed haplotypes) and one dataset including only native (non-introgressed) haplotypes. Native and introgressed haplotypes in the sampling were identified after Vollmer and Palumbi (2002, 2007) [21], [32]. Significance of differences in introgression frequencies between populations was compared using a G-test of independence [58].

### Population Genetic Statistics

DNA sequence polymorphism for each population was characterized using DnaSP 4.0 [59]. A Statistical Parsimony Network was constructed in TCS version 1.21 [60]. Haplotypes were identified as shared between two or more populations or as unique to a single population (private haplotypes), and introgression frequencies were calculated for each population as the percentage of haplotypes sampled identified as originating in the *A. palmata* lineage. Using the native haplotypes we tested for deviations from neutral expectations using standard tests: Tajima's D [61], Fu and Li's D [62], and Fu and Li's F [62]. In addition, we ran mismatch analyses to detect signatures of population expansion or contraction against the null hypothesis of a constant-sized population and used coalescent simulations to test the significance of population size changes using the R_2_ statistic [63].

### Population Genetic Structure

Hierarchical Analysis of Molecular Variance (AMOVA) [64] was conducted using Arlequin 2.0 [65] to test for population genetic structure among four regions across the seven sampled populations. The regions were defined as follows: Western Caribbean (Belize and Panama), Eastern Caribbean (Curacao and Puerto Rico), Greater Bahamas (Bahamas and Turks and Caicos), and Florida. To estimate genetic structure between populations, pairwise F_ST_ (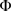
_ST_) values were calculated between each population. Significance was determined by 10,100 permutations and *P*-values were adjusted using sequential Bonferroni [66]. Genetic structure between regions within the Florida Keys (as stated above) was evaluated by calculating F_ST_.
